# Transcriptome- and Metabolome-Based Regulation of Growth, Development, and Bioactive Compounds in *Salvia miltiorrhiza* (Lamiaceae) Seedlings by Different Phosphorus Levels

**DOI:** 10.3390/ijms26136253

**Published:** 2025-06-28

**Authors:** Kewei Zuo, Lingxing Chen, Tian Li, Shuang Liu, Chenlu Zhang

**Affiliations:** 1 School of Biological Science and Engineering, Shaanxi University of Technology, Hanzhong 723000, China; keweizuo@163.com (K.Z.); 18829453924@163.com (L.C.); litian202212@163.com (T.L.); liushuang3680@163.com (S.L.); 2 Collaborative Innovation Center for Comprehensive Development of Biological Resources in Qinba Mountainous Area, Hanzhong 723000, China; 3 Shaanxi Provincial Key Laboratory of Resource Biology, Hanzhong 723000, China; 4 State Key Laboratory of Biological Resources and Ecology and Environment, School of Biology, School of Biological Science and Engineering, South Campus, Shaanxi University of Technology, Hanzhong 723000, China

**Keywords:** phosphorus, *Salvia miltiorrhiza* Bunge, secondary metabolism, transcription factors, metabolomics

## Abstract

Phosphorus (P), as one of the essential bulk elements for plant growth and development, plays an important role in root growth, accumulation of secondary metabolites, and regulation of gene expression. In *Salvia miltiorrhiza* Bunge (*S. miltiorrhiza*), an important medicinal plant, the accumulation of its active components is closely related to the level of phosphorus supply, but the molecular regulatory mechanism of phosphorus treatment in the growth and secondary metabolism of *S. miltiorrhiza* is not clear. In this study, we investigated the effects of low phosphorus (P2), moderate phosphorus (P4), and high phosphorus (P6) treatment on the growth and development of *S. miltiorrhiza*. seedlings, the accumulation of bioactive compounds, and their transcriptional regulation using transcriptomic and metabolomic analyses, and identified the key regulatory genes in the biosynthesis pathways of tanshinone and salvianolic acid. The findings revealed that *S. miltiorrhiza* biomass exhibited a “peaked” response to phosphorus concentration, peaking at 0.625 mmol·L^−1^. At this optimal concentration, all four batches achieved maximum root length, root weight, and leaf weight: Batch I (11.3 cm, 2.34 g, 1.62 g), Batch II (12.7 cm, 2.67 g, 1.89 g), Batch III (13.8 cm, 2.85 g, 2.04 g), and Batch IV (15.6 cm, 3.51 g, 2.44 g). Both lower and higher concentrations resulted in growth inhibition and reduced bioactive compound accumulation. Transcription factors associated with root growth and development included bHLH, MYB, and WRKY; in particular, the bZIP23 transcription factor was highly expressed under abnormal phosphorus supply conditions. In addition, the biosynthetic pathways of tanshinone and salvianolic acid were elucidated, and key genes related to the synthesis pathways (CPS, KSL, CYP, PAL, HPPR, and RAS) were identified. The expression of several TFs (such as SmCPS1, SmCYP76AH3, SmCYP76AH1, SmGGPPS1, and SmRAS1) was found to be correlated with tanshinone and salvianolic acid synthesis. The present study provides a theoretical basis for further revealing the molecular mechanism of phosphorus regulation of growth, development, and secondary metabolism of *S. miltiorrhiza* and provides potential targets for efficient cultivation and molecular breeding of *S. miltiorrhiza.*

## 1. Introduction

*Salvia miltiorrhiza* Bunge (*S. miltiorrhiza*) is a perennial herb from the Lamiaceae family with roots that are used for medicinal purposes. The roots possess several efficacy properties, such as activating blood circulation, removing blood stasis, clearing the heart, removing vexation, cooling the blood, and eliminating pain [1]. Its medicinal value was first recorded in Shennong Ben Cao Jing (Classic of the Materia Medica of the Divine Husbandman) and was classified as a top-quality product with a wide range of pharmacological effects, including anti-arrhythmia, regulation of blood lipids, anti-atherosclerosis, improvement of microcirculation, and protection of cardiomyocytes. The root of *S. miltiorrhiza* is its main medicinal part, and its bioactive compounds are specifically distributed in the root tissue. It was found that the content of each component in the root of *S. miltiorrhiza* gradually decreased from the outside to the inside, and the highest content was found in the epidermis. Additionally, the content of water-soluble components showed a decreasing trend while the content of fat-soluble components showed an increasing trend from the top to the bottom of the root. In addition, the morphological structure of the root system of *S. miltiorrhiza*, especially the number of divided roots, had a significant effect on the content of active components in the root system of a single plant [2].

Phosphorus is an essential bulk element for plant growth and development, crucial for physiological metabolism and secondary metabolite accumulation. However, soil phosphorus has low metastability, making it hard for plants to utilize effectively and potentially inhibiting growth [3]. Studies show different phosphorus concentrations significantly affect secondary metabolite accumulation in plants. In windbreak (*Saposhnikovia divaricata*), moderate phosphorus treatments increase soil effective phosphorus, promoting secondary metabolite accumulation. Phosphorus deficiency can limit nutrient uptake and affect secondary metabolite synthesis. The accumulation of these metabolites is closely related to the plant’s growth environment, and insufficient phosphorus may alter metabolic pathways, impacting the types and amounts of secondary metabolites [4,5]. Thus, rationally applying phosphorus fertilizer in agriculture is vital for enhancing secondary metabolite accumulation in plants. For medicinal plants like *S. miltiorrhiza*, appropriate phosphorus supply can improve their medicinal value by regulating root system structure and bioactive compound distribution.

Joint multi-omics analyses provide a new perspective and approach for in-depth understanding of the complex mechanisms of secondary metabolic accumulation in medicinal plants [6,7]. By integrating multi-omics data such as transcriptomics, metabolomics, and proteomics, the metabolic regulatory network of medicinal plants under different environmental conditions can be more comprehensively analyzed; this approach has been widely used in various fields, such as traditional Chinese medicine, pathology, and agronomy [8,9,10]. In the study of *S. miltiorrhiza*, the potential mechanism of Salvianolic acid A biosynthesis induced by UV-B irradiation was successfully resolved by integrating metabolomics, proteomics, and transcriptomics data based on a multi-omics approach [11]. Additionally, in the study of the regulatory mechanism of tanshinone biosynthesis, SmWRKY32 was found to negatively regulate tanshinone biosynthesis by directly inhibiting GGPPS1, CPS1, and ERF128, thereby revealing a new biosynthesis pathway for tanshinone regulation [12]. Joint multi-omics analysis can not only reveal the biosynthetic pathways of secondary metabolites in medicinal plants, but also provide a scientific basis for improving the medicinal value of medicinal plants and developing new drug resources.

In this study, we employed *S. miltiorrhiza* seedlings grown under varying phosphorus supply conditions to explore how phosphorus regulates root growth, development, and secondary metabolism, as well as the underlying transcriptional mechanisms. We applied phosphorus concentration gradients (ranging from 0.0156 mmol·L^−1^ to 10 mmol·L^−1^) and evaluated the resulting growth performance, focusing on morphological traits and biomass distribution in both aboveground and belowground parts. Using HPLC and other analytical techniques, we measured the levels and composition of key secondary metabolites, such as tanshinones and salvianolic acid. At the molecular level, we integrated transcriptomic and metabolomic analyses to identify differentially expressed genes and metabolic pathways responsive to phosphorus. By examining the expression patterns of genes involved in phosphorus and secondary metabolism, we uncovered transcriptional regulatory networks. Our findings enhance the understanding of phosphorus’s role in *S. miltiorrhiza*’s physiology, offering strategies to optimize cultivation practices, boost medicinal compound yields, and identify targets for molecular breeding of high-yielding *S. miltiorrhiza* varieties.

## 2. Results

### 2.1. Effects of Different Levels of Phosphorus Supply on the Growth Pattern of S. miltiorrhiza Seedlings

**Figure 1:**
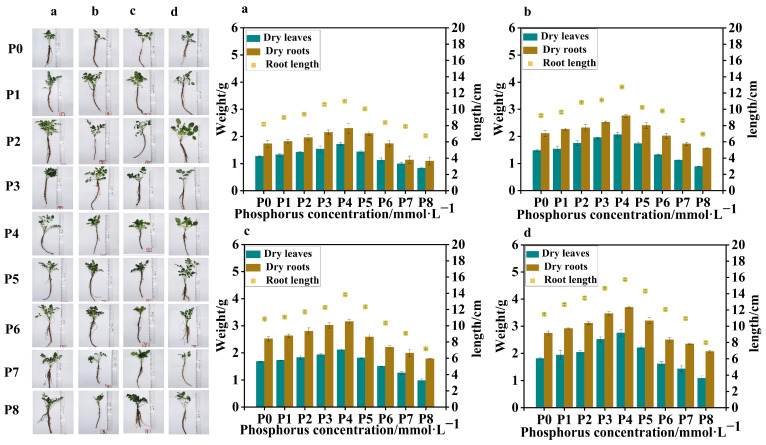
Statistics on the differences in growth phenology and morphology of four batches of *S. miltiorrhiza* seedlings collected at different points of time. Panels (**a**–**d**) represent four batches of samples collected at different time points, while P0 to P8 denote different levels of phosphorus supply. Bar graphs of different colors indicate the weight of dried roots and dried leaves, and scatter plots indicate root length.

Four batches of samples were obtained from *S. miltiorrhiza* seedlings treated with different levels of phosphorus supply on four occasions, with an interval of 7 days between each sampling. Compared to the control group (P0), in the samples collected from the four batches, the low-phosphorus group showed similar data for dry leaf weight, dry root weight, and root length, with no obvious variation. However, in the high-phosphorus group, there was a significant variation in dry leaf weight, dry root weight, and root length between the groups, with clear trends. At the same time, in the 4th and 3rd batch samples, due to the longer duration of phosphorus treatment, all groups showed significant differences in dry leaf weight, dry root weight, and root length compared to the 1st and 2nd batch samples. The leaf weight of the high-phosphorus group increased at the beginning, but its growth rate gradually slowed down with time, and the root system was short, thick, less branched, and the overall root structure was not as well developed as that of the low-phosphorus group (Figure 1).

In all batches of samples, leaf weight, root weight, and root length of *S. miltiorrhiza* seedlings in the low-phosphorus group were relatively higher than those of *S. miltiorrhiza* seedlings in the high-phosphorus group. However, the growth of *S. miltiorrhiza* seedlings treated with P7 (5 mmol·L^−1^) and P8 (10 mmol·L^−1^) was seriously inhibited, with slow stem and leaf growth, fewer and slender lateral roots, and shortening of the main root, suggesting that too high a concentration of phosphorus inhibited the growth of *S. miltiorrhiza* seedlings, and at the same time, the biomass did not reach the experimental demand, so the late composition was not analyzed further. Interestingly, the overall trends of leaf weight, root weight, and root length of *S. miltiorrhiza* seedlings were positively correlated in the four samplings (Figure 1).

The trends of root length, root weight, and leaf weight of *S. miltiorrhiza* at different phosphorus supply concentrations were peaked, reaching the highest with the P4 (0.625 mmol·L^−1^) treatment. The root lengths of the low- and high-phosphorus group treatments were significantly lower than those of the P4 treatment in the following order: P4 (0.625 mmol·L^−1^) > P3 (0.313 mmol·L^−1^) > P5 (1.25 mmol·L^−1^) > P2 (0.0313 mmol·L^−1^) > P1 (0.0156 mmol·L^−1^) > P0 (0 mmol·L^−1^) > P6 (2.5 mmol·L^−1^) > P7 (5 mmol·L^−1^) > P8 (10 mmol·L^−1^). Moreover, the effects of different phosphorus supply levels on the root and leaf weights of *S. miltiorrhiza* seedlings exhibited distinct patterns. The overall trend for both root and leaf weights showed an initial increase followed by a decrease, with the highest values observed at moderate phosphorus concentrations. In the range of 0.0~0.625 mmol·L^−1^, both root weight and leaf weight increased with the increase in phosphorus concentration, and in the range of 0.625~10 mmol·L^−1^, both root weight and leaf weight decreased with the increase in phosphorus concentration.

In conclusion, the environmental optimum phosphorus concentration was 0.625 mmol·L^−1^, and appropriately lowering or elevating the phosphorus supply level did not have much effect on the root growth and development of *S. miltiorrhiza*, but both low and high phosphate concentrations significantly inhibited the growth of *S. miltiorrhiza*.

### 2.2. Determination of the Content of Active Components in the Root Systems of S. miltiorrhiza Seedlings Grown Under Different Levels of Phosphorus Supply

**Figure 2:**
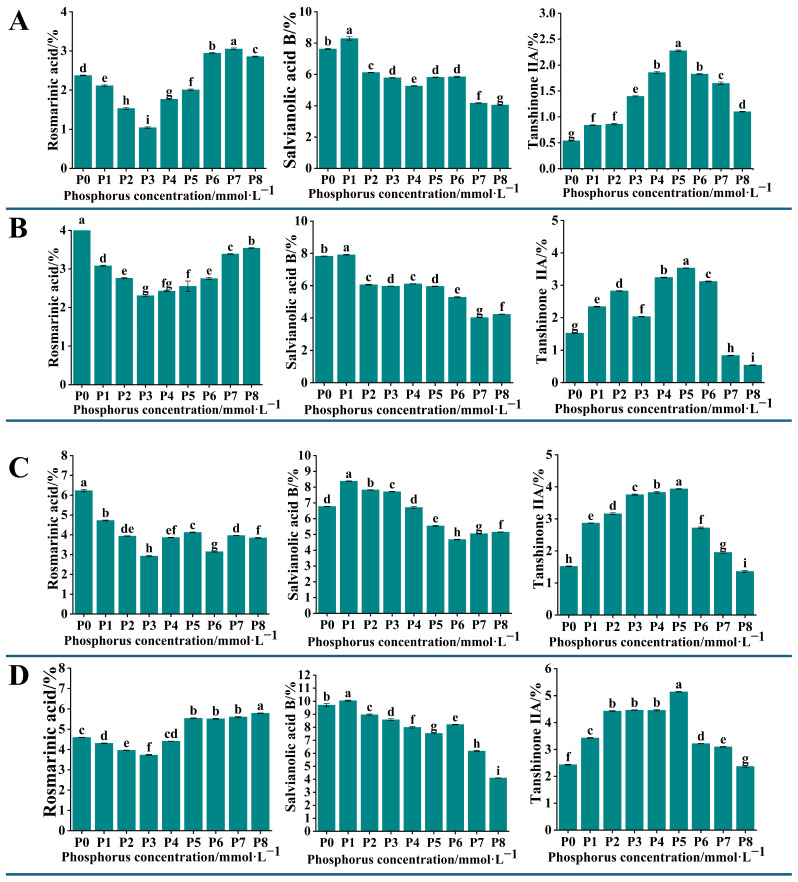
Changes in the content of bioactive compounds in the roots of *S. miltiorrhiza* seedlings across four sampling batches under different phosphorus treatments. Panels (**A**–**D**) illustrate the differences in the content of rosmarinic acid, salvianolic acid B, and tanshinone IIA in the roots of *S. miltiorrhiza* seedlings from the first, second, third, and fourth sampling batches, respectively. Significant differences between groups are indicated by the letters a, b, c, and d. Groups sharing the same letter are not significantly different (*p* > 0.05), while groups with different letters show significant differences (*p* < 0.05). The Y-axis represents the content of each compound as a percentage of the dry weight of the sample (% *w*/*w*).

To detect the changes in the content of active components in the root systems of *S. miltiorrhiza* seedlings grown under different levels of phosphorus supply, we analyzed the content of 11 active components in *S. miltiorrhiza* roots. The HPLC chromatogram presented in Figure S1 represents a mixed control solution of these active components. The results showed that the contents of rosmarinic acid, salvianolic acid B, and tanshinone IIA exhibited significant changes (*p* < 0.05). The content of rosemarinic acid in *S. miltiorrhiza* roots showed a decreasing and then increasing trend, and reached a minimum at a phosphorus concentration of 0.313 mmol·L^−1^ (P3). The content of salvianolic acid B showed a decreasing and then stabilizing trend with the increase in phosphorus concentration, and reached the maximum at the phosphorus concentration of 0.0156 mmol·L^−1^ (P1). The trend of tanshinone IIA content increased with the increase in phosphorus concentration; the content was highest at 1.25 mmol·L^−1^ (P5) and decreased continuously at phosphorus concentrations higher than P5 (Figure 2).

The above results indicated that moderate low phosphorus conditions could promote the growth of roots as well as the accumulation of secondary metabolites in *S. miltiorrhiza* seedlings. Since the fourth batch of collected samples had sufficient time for phosphorus treatment, the growth morphology of *S. miltiorrhiza* seedlings and the accumulation of active components in roots within the group varied greatly under different phosphorus treatments, so the fourth batch of collected samples was selected for analysis in the subsequent transcriptome sequencing.

### 2.3. Transcriptomics Analysis

The 12 samples (P0_a to P6_c) used for transcriptome sequencing were obtained from the root system of *S. miltiorrhiza* seedlings subjected to different phosphorus treatments. Specifically, P0 represents the control group, while P2, P4, and P6 represent seedlings treated with low, medium, and high phosphorus concentrations, respectively. The suffixes a, b, and c denote three biological replicates for each treatment group. Total RNA was extracted from the root system of these seedlings. Transcriptome sequencing of these RNA samples generated a total of 73.13 Gb of clean data, with each sample yielding over 5.94 Gb of clean data. The Q30 percentages of the 12 samples P0_a to P6_c selected from different phosphorus supply levels for treating *S. miltiorrhiza* seedlings in this experiment ranged from 92.48% to 93.38%, which was much higher than the standard value of 80%, and the percentages of G and C in the nine samples accounting for the four bases were around 52%, with a close base content (Table S1). Therefore, the analysis of the sequencing quality results for *S. miltiorrhiza* showed that the obtained sequencing data were reliable and complete, and could be used for further data mining and bioinformatics analysis.

**Figure 3:**
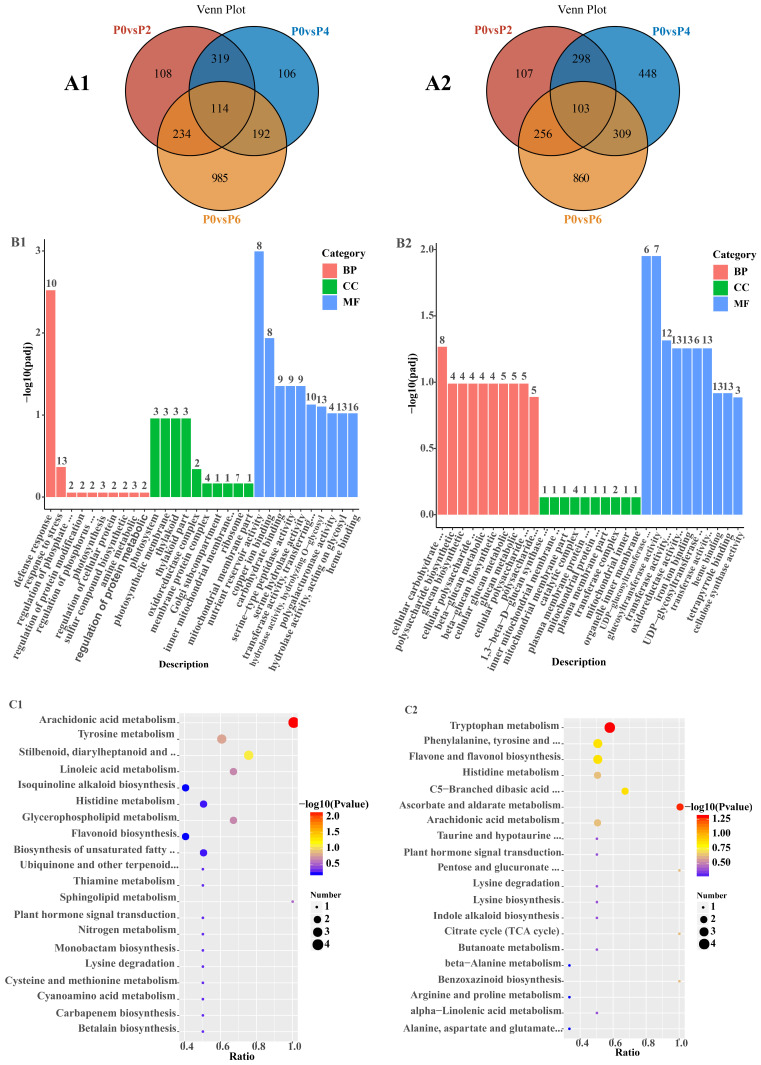
Venn diagrams of up-regulated and down-regulated differentially expressed genes, GO enrichment analysis and KEGG enrichment analysis. Panels (**A1**,**A2**) denote the Venn diagrams of up- and down-regulated DEGs in the three comparison groups, with different colors indicating DEGs from different comparison groups. Panels (**B1**,**B2**) denote the results of GO enrichment analysis for up-regulated and down-regulated DEGs, respectively, with different colored bar graphs indicating the number of genes enriched in biological processes (BP), cellular components (CC), and molecular functions (MF). Panels (**C1**,**C2**) denote the results of KEGG pathway enrichment analysis for up-regulated and down-regulated DEGs, respectively. The size of the dots indicates the number of genes associated with the pathway, and the color of the dots indicates the *p*-value, with red indicating higher significance. The ‘ratio’ in (**C1**,**C2**) refers to the proportion of differentially expressed genes (DEGs) enriched in a specific pathway relative to the total number of genes annotated in that pathway within the reference genome.

The statistical results for DEGs in Salvia seedlings between different comparison groups are shown in (Table S2). Among the 6481 detected DEGs, the number of up-regulated DEGs under low phosphorus conditions increased from 775 to 1525 under high phosphorus conditions, and the number of down-regulated DEGs under low phosphorus conditions increased from 764 to 1528 under high phosphorus conditions. Among the 3031 up-regulated DEGs, 114 common DEGs were detected in P0 vs. P2, P0 vs. P4, and P0 vs. P6, and 108, 106, and 985 specific DEGs were detected in P0 vs. P2, P0 vs. P4, and P0 vs. P6, respectively (Figure 3(A1)). Among the 3450 down-regulated DEGs, 103 common DEGs were detected in P0 vs. P2, P0 vs. P4, and P0 vs. P6, and 107, 448, and 860 specific DEGs were detected in P0 vs. P2, P0 vs. P4, and P0 vs. P6, respectively (Figure 3(A2)).

GO analysis of 114 up-regulated DEGs showed (Figure 3(B1)) that these DEGs were predominantly enriched in defense response processes, photosynthesis, and nutrient storage pools in the biological process (BP), cell component (CC), and molecular function (MF) categories, respectively. KEGG analysis of 114 up-regulated DEGs showed (Figure 3(C1)) that these DEGs were predominantly enriched in metabolic pathways, secondary metabolite biosynthesis, and phytohormone signaling. GO analysis of 103 down-regulated DEGs showed (Figure 3(B2)) that these DEGs were mainly enriched in cellular carbohydrate metabolism processes, mitochondrial inner membrane complexes, and glucosyltransferase activities in the biological process (BP), cellular component (CC), and molecular function (MF) categories, respectively. The KEGG analysis of 103 down-regulated KEGG analysis of DEGs showed (Figure 3(C2)) that these DEGs were predominantly enriched in multiple metabolic pathways, lysine degradation, and glucuronide conversion pathways.

The results of GO analysis of up-/down-regulated DEGs in P0 vs. P2, P0 vs. P4, and P0 vs. P6 only showed (Tables S3 and S4) that in the BP category, up-regulated DEGs in P0 vs. P2 were mainly enriched in translational processes, up-regulated DEGs in P0 vs. P4 were mainly enriched in amide biosynthesis processes, and up-regulated DEGs in P0 vs. P6 were mainly enriched in ketone metabolic and defense processes. Down-regulated DEGs in P0 vs. P2 were mainly enriched in secondary metabolite metabolism processes, down-regulated DEGs in P0 vs. P4 were mainly enriched in inorganic cation transport processes, and down-regulated DEGs in P0 vs. P6 were mainly enriched in sugar metabolism and small molecule catabolic processes.

In the CC category, up-regulated DEGs in P0 vs. P2 were mainly enriched in ribosomes and nucleic acid–protein complex fractions, up-regulated DEGs in P0 vs. P4 were mainly enriched in membrane-free organelles, and up-regulated DEGs in P0 vs. P6 were mainly enriched in the membranes of photosynthesizing organelles. Down-regulated DEGs in P0 vs. P2 were mainly enriched in the periphery of the cell, down-regulated DEGs in P0 vs. P4 were mainly enriched in the Golgi, and down-regulated DEGs in P0 vs. P6 were mainly enriched in the outer cortex part of the cell.

In the MF category, DEGs up-regulated in P0 vs. P2 were mainly enriched in ion transmembrane transport protein activity, DEGs up-regulated in P0 vs. P4 were mainly enriched in ribosomal structural component activity, and DEGs up-regulated in P0 vs. P6 were mainly enriched in DNA-binding transcription factor activity. Down-regulated DEGs in P0 vs. P2 were mainly enriched in hydrolase activity, down-regulated DEGs in P0 vs. P4 were mainly enriched in GTPase binding activity, and down-regulated DEGs in P0 vs. P6 were mainly enriched in oxidoreductase activity.

KEGG analysis showed (Table S5) that there were two common pathways in which P0 vs. P2, P0 vs. P4, and P0 vs. P6-specific DEGs were enriched, i.e., the metabolic pathway and the biosynthesis pathway of secondary metabolites. DEGs up-regulated in P0 vs. P2 were mainly enriched in ubiquinone and other terpenoid quinone biosynthesis, those up-regulated in P0 vs. P4 were mainly enriched in flavonoid and brassinol biosynthesis and sesquiterpene and triterpene biosynthesis, and those up-regulated in P0 vs. P6 were mainly enriched in amino acid biosynthesis and biotin metabolism. DEGs down-regulated in P0 vs. P2 were mainly enriched in starch and sucrose metabolism, those down-regulated in P0 vs. P4 were mainly enriched in lysine degradation metabolism and fructose and mannose metabolism, and those down-regulated in P0 vs. P6 were mainly enriched in pentose phosphate pathway and oxidative phosphorylation metabolic process.

**Figure 4:**
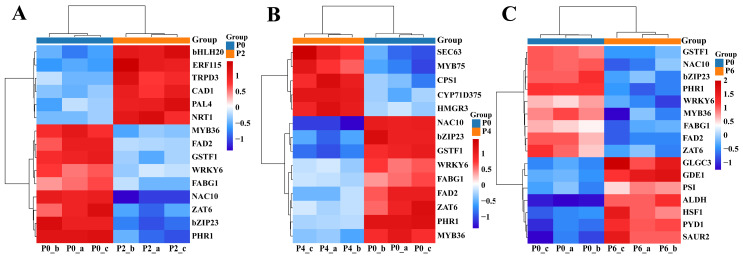
Heatmaps of DEG clustering. Panels (**A**–**C**) denote heatmaps analyzed by P2 vs. P0, P4 vs. P0, and P6 vs. P0 screening DEGs, respectively. Red indicates a higher level of gene expression (up-regulation). Blue indicates a lower level of gene expression (down-regulation). White indicates no significant change in gene expression level. The heatmaps display the gene expression changes related to phosphate uptake and transport, bioactive compound accumulation, and root growth and development under P0, P2, P4, and P6 treatments. Color shades indicate the relative high or low level of gene expression.

In order to investigate the genes that responded to the regulation of root growth and development and root bioactive compound metabolism in *S. miltiorrhiza* seedlings under different levels of phosphorus supply, DEGs were analyzed, and genes related to phosphate uptake and transport, bioactive compound accumulation, and root growth and development were screened for heatmaps (Figure 4). As can be seen in Figure 4, six up-regulated genes were retrieved in the root transcriptome at P2 compared to P0, namely protein transcription factor superfamily member 20 (bHLH20), transcription factor of the AP2/ERF family (ERF115), tryptophan manipulator (TRPD3), cinnamyl alcohol dehydrogenase (CAD1), gene of key enzyme of phenylpropanoid metabolic pathway (PAL4), and nitrate transporter protein (NRT1).

Five up-regulated genes were retrieved in the root transcriptome at P4 compared to P0, namely protein transporter-related genes (SEC63), MYB family transcription factor member 75 (MYB75), key enzyme genes of the tanshinone secondary metabolic pathway (CPS1), key enzyme genes for the biosynthesis of tanshinone compounds (CYP71D375), and key enzyme gene for the biosynthesis of tanshinone constituents (HMGR3).

Seven up-regulated genes were retrieved from the root transcriptome at P6 compared to P0, namely recombinant adenosine diphosphate glucose pyrophosphorylase gene (GLGC3), glycerol dihydrogen phosphate phosphodiesterase 1 (GDE1), phosphate starvation inducible gene (PSI), aldehyde dehydrogenase superfamily (ALDH), phosphorus treatment-related response gene (HSF1), Saccharomyces cerevisiae surface display vector (PYD1), and growth hormone up-regulated small RNA (SAUR2). Notably CYP71D375, CPS1, and HMGR3, transcription factors associated with the regulation of tanshinone synthesis, were highly expressed in the P4 condition, and bHLH20, PAL4, and ERF115, transcription factors associated with the regulation of tanshinone synthesis, were highly expressed in the P2 condition.

**Figure 5:**
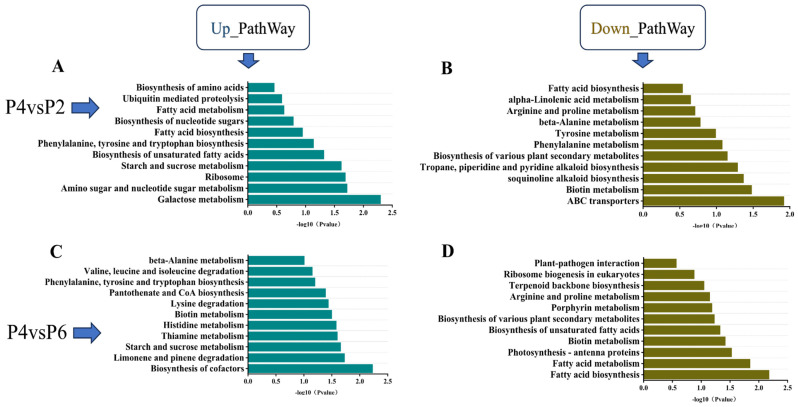
KEGG pathway enrichment. Panels (**A**,**B**) show the up-regulated and down-regulated pathways under P4 vs. P2 conditions, respectively. Panels (**C**,**D**) show the up-regulated and down-regulated pathways under P4 vs. P6 conditions, respectively. The horizontal coordinate is −log10 (*p*-value), which indicates the significance level of pathway enrichment, with larger values indicating more significant pathway enrichment.

KEGG pathway enrichment analysis was conducted for the low-phosphorus (P2), medium-phosphorus (P4), and high-phosphorus (P6) groups, with results presented in Figure 5. The analysis revealed that the pathways of phenylalanine, tyrosine, and tryptophan biosynthesis, as well as starch and sucrose metabolism, were co-enriched in the comparisons of P2 vs. P4 and P6 vs. P4. Specifically, both low-phosphorus and high-phosphorus conditions led to significant up-regulation of genes in the starch and sugar metabolism pathways of *S. miltiorrhiza* seedlings. Furthermore, when compared to the medium-phosphorus group (P4), the phenylalanine, tyrosine, and tryptophan biosynthesis pathways exhibited significantly higher levels of gene expression (Figure 5A,C). The co-enriched down-regulated pathways included fatty acid biosynthesis, arginine and proline metabolism, biosynthesis of various plant secondary metabolites, and biotin metabolism. Compared with the medium-phosphorus group P4, both the low-phosphorus and high-phosphorus environments resulted in a significant down-regulation of the expression levels of genes regulating the above four pathways in *S. miltiorrhiza* seedlings, but the down-regulation of genes regulating fatty acid biosynthesis in the high-phosphorus environment was lower compared with the medium-phosphorus environment, which regulated fatty acid biosynthesis more significantly (Figure 5B,D).

### 2.4. Analysis of DEGs and TFs Related to the Synthetic Pathway of Tanshinone and Salvianolic Acid

#### 2.4.1. DEGs Associated with Tanshinone Biosynthesis

**Figure 6:**
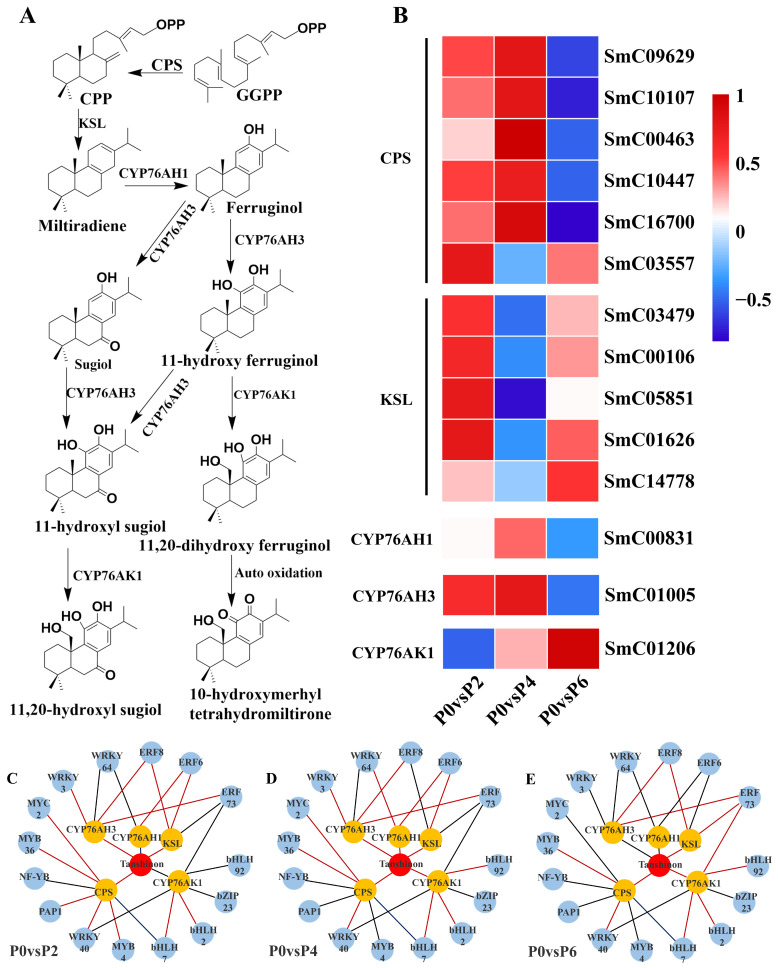
Expression levels of structural genes for tanshinone biosynthesis at different phosphorus supply levels and their correlation between TFs and tanshinones. (**A**) Tanshinone biosynthesis pathway in the root system of *S. miltiorrhiza*. (**B**) Genes involved in the tanshinone biosynthesis pathway. (**C**–**E**) TF regulatory network for tanshinone biosynthesis. In (**C**–**E**), blue, orange, and red colors indicate TFs, genes, and tanshinones, respectively; red and black lines indicate positive and negative correlations, respectively.

Fourteen DEGs were found to be associated with the tanshinone synthesis pathway (Figure 6A), and among the 14 DEGs, five single genes encoding cobalt pyrophosphate synthase (CPS) (SmC09629, SmC10107, SmC00463, SmC10447, and SmC16700) were up-regulated in the P4 condition compared to the P0 group; two single genes encoding cytochrome P450 enzymes (CYP761AH1 and CYP761AH3) were up-regulated in the P4 condition; and the expression of the five single genes encoding kaurane synthase (KSL) (SmC03479, SmC00106, SmC05851, SmC01626, SmC14778) was up-regulated. Notably, CYP761AK1, CYP761AH1, and CYP761AH3, encoding cytochrome P450 enzymes, were not equally expressed under P2, P4, and P6 conditions, with the expression of CYP761AK1 being suppressed under low phosphorus conditions, and the expression of CYP761AH1 and CYP761AH3 being suppressed under high phosphorus conditions (Figure 6B).

#### 2.4.2. DEGs Associated with Salvianolic Acid Biosynthesis

**Figure 7:**
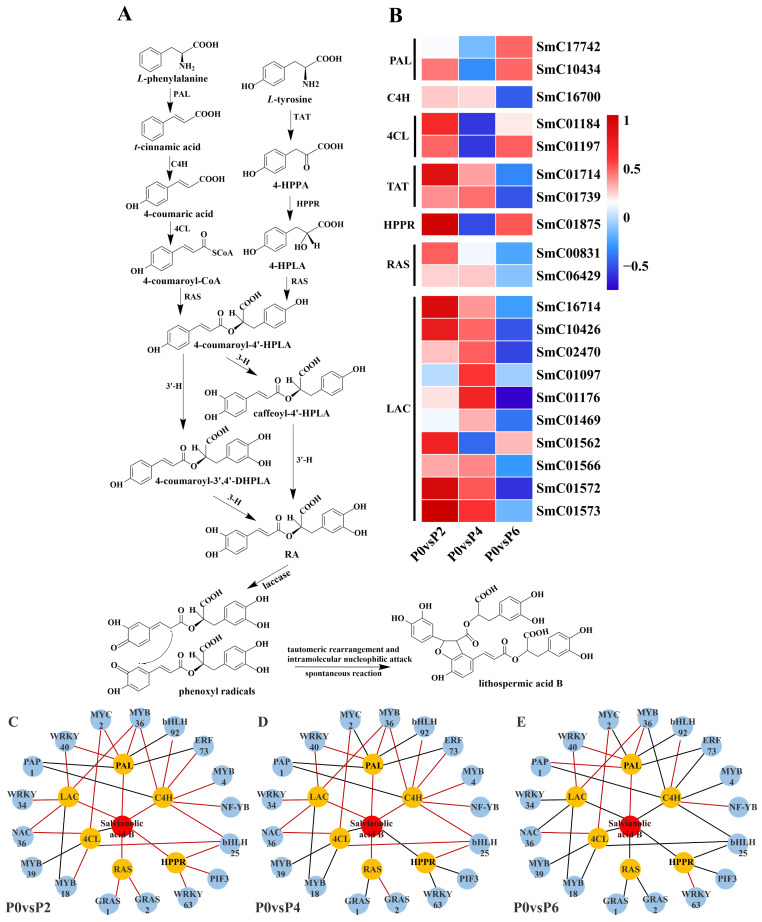
Expression levels of structural genes for salvianolic acid biosynthesis at different levels of phosphorus supply and their correlation between TFs and salvianolic acid. (**A**) Salvianolic acid biosynthesis pathway in the root system of *S. miltiorrhiza*. (**B**) Genes involved in the salvianolic acid biosynthesis pathway. (**C**–**E**) TF regulatory network for salvianolic acid biosynthesis. In (**C**–**E**), blue, orange, and red colors indicate TFs, genes, and salvianolic acid, respectively; red and black lines indicate positive and negative correlations, respectively.

Twenty DEGs were found to be associated with the synthesis pathway of salvianolic acid (Figure 7A), and among these 20 DEGs, compared to the P0 group, the expression of two single genes (SmC17742 and SmC10434) encoding phenylalanine ammonia-lyase (PAL), two single genes (SmC01184 and SmC01197) encoding 4-coumarin CoA ligase (4CL), and one single gene (SmC01875) encoding *p*-hydroxyphenylpyruvate reductase (HPPR) were up-regulated in P2 and P6 conditions and suppressed in P4 conditions. One single gene (SmC16700) encoding cinnamate-4-hydroxylase (C4H), two single genes (SmC01714 and SmC01739) encoding tyrosine aminotransferase (TAT), two single genes (SmC00831 and SmC06429) encoding rosemarinic acid synthetase (RAS), and eight single genes (SmC16714 and SmC06429) encoding laccase genes (SmC16714, SmC10426, SmC02470, SmC01176, SmC01469, SmC01566, SmC01572, and SmC01573) were up-regulated in P2 and P4 conditions, and suppressed in P6 conditions. Notably, most of these 20 detected DEGs associated with the salvianolic acid synthesis pathway were up-regulated in low phosphorus conditions (P2) in terms of gene expression (Figure 7B).

#### 2.4.3. TFs Related to the Biosynthesis of Tanshinone and Salvianolic Acid

The results showed that 15 TFs were associated with the expression of tanshinone biosynthesis pathway genes (Figure 6C–E), and 17 TFs were associated with the expression of genes in the salvianolic acid biosynthesis pathway (Figure 7C–E). In the tanshinone biosynthesis pathway, the expression levels of genes involved in tanshinone biosynthesis were up-regulated under P4 conditions, with most of the associated TFs being positively regulated. In contrast, the tanshinone synthesis pathway was partially inhibited under P6 and P2 conditions, with the TFs being negatively regulated. In the salvianolic acid biosynthesis pathway, the expression levels of genes involved in salvianolic acid biosynthesis were up-regulated under P2 and P4 conditions, with most TFs positively regulated. However, under P6 conditions, the salvianolic acid synthesis pathway was inhibited, and the TFs were negatively regulated. Notably, in both biosynthetic pathways, the expression of MYB and WRKY transcription factors was up-regulated under P2 and P4 conditions and down-regulated under high-phosphorus conditions. These transcription factors are known to influence the synthesis of tanshinone and salvianolic acid by regulating the expression of key enzyme genes in secondary metabolic pathways.

### 2.5. qRT-PCR Validation

**Figure 8:**
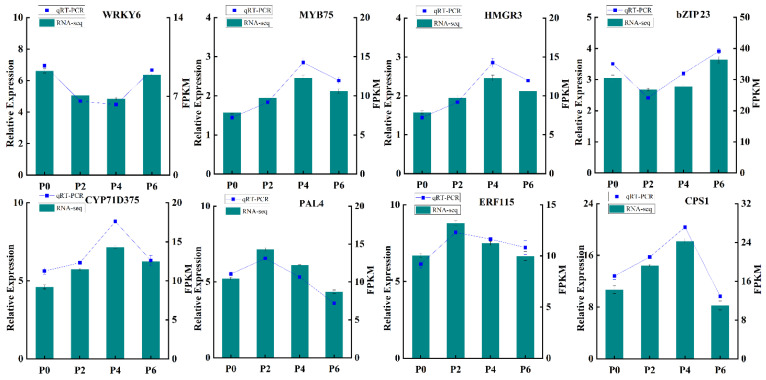
qRT-PCR validation. The gene expression trends detected by the two methods were consistent, validating the reliability of the RNA-seq data.

In order to further verify the reliability and precision of the transcriptome sequencing results, eight differentially expressed genes were carefully selected for qRT-PCR validation (Figure 8). The qRT-PCR analysis was conducted using specific primers designed for these genes, ensuring the accuracy of the measurement. The results of the qRT-PCR analysis demonstrated a remarkable consistency between the measured expression levels of these genes across different groups and the FPKM values obtained from the RNA-Seq data. This congruence provided robust evidence that the transcriptome data were highly reliable and could accurately reflect the effects of varying phosphorus supply levels on the growth, development, and gene expression of *S. miltiorrhiza* seedlings. Thus, the results of this validation step strengthen the credibility of the entire study and pave the way for further exploration into the molecular mechanisms underlying the response of *S. miltiorrhiza* to different phosphorus conditions.

## 3. Discussion

At present, the lack of available phosphorus resources in soil has become one of the global agricultural challenges [13,14], and it is of great practical significance to study the effects of phosphorus supply levels on plant growth and development processes [15].

It has been previously shown that under phosphorus treatment conditions, *Arabidopsis thaliana* alters the development of lateral roots by regulating the growth hormone signaling pathway, which in turn affects phosphorus uptake and utilization in plants [16]. Similarly, the tolerance of hairy roots of *S. miltiorrhiza* to phosphorus treatment has been linked to secondary metabolism and antioxidant defenses [17], as well as the genome-wide identification and characterization of the *S. miltiorrhiza* PHT1 gene family and its response to mycorrhizal symbiosis under phosphate treatment [18].These findings reveal the ability of plants to maintain growth and development by regulating internal signaling pathways and metabolic processes to adapt to environmental changes in the face of phosphorus treatment.

The changes in the content of 11 active components in the roots of *S. miltiorrhiza* transplant seedlings under different phosphorus supply levels were analyzed by HPLC. The results showed significant changes in the content of rosmarinic acid, salvianolic acid B, and tanshinone IIA. The content of rosmarinic acid initially decreased and then increased with the increase in phosphorus concentration, reaching the lowest point at a phosphorus concentration of 0.313 mmol·L^−1^. Its variation may be related to the fluctuation in the content of caffeic acid and salvianolic acid, and there may be a positive synthetic relationship between them [19]. The content of salvianolic acid B was similar to that of lithospermic acid and reached its maximum at a phosphorus concentration of 0 mmol·L^−1^, which was significantly higher than that of the other treatment groups, and its changes were closely related to the changes in the content of caffeic acid and lithospermic acid. The content of tanshinone IIA was the highest at a phosphorus concentration of 1.25 mmol·L^−1^, and gradually decreased with the increase in phosphorus concentration (Figure 2).

In conclusion, the environmental optimum phosphorus concentration was 0.625 mmol·L^−1^ (Figure 1). Appropriate reduction or elevation of the phosphorus supply level did not have much effect on the root growth and development of *S. miltiorrhiza*, but both too low and too high phosphorus concentrations inhibited plant growth and development as well as the accumulation of secondary metabolites.

The molecular mechanisms of root growth, development, and secondary metabolism of *S. miltiorrhiza* seedlings treated with different phosphorus supply levels were further analyzed by transcriptomics and metabolomics. Transcriptomics revealed that bZIP23, a major regulator controlling various response processes of plants to external stimuli [20], was highly expressed in both the low phosphorus level-treated group P2 and high phosphorus level-treated group P6, and by interacting with the cis-acting elements in the promoter region of the PHT1 gene, it regulated the transcription level of the target genes and, consequently, the treatment tolerance of *S. miltiorrhiza* [17]. In response to the low phosphorus environment, the PHR transcription factor binds to the P1BS element of the promoter of the low phosphorus responsive genes to activate the expression of low phosphorus-responsive genes and increase phosphorus uptake by the root system of *S. miltiorrhiza* [21]. The NRT gene is activated to promote nitrate uptake and to regulate the nitrogen metabolism in order to compensate for growth inhibition caused by phosphorus deficiency [22]. Meanwhile, transcription factors such as NAC10, GSTF1, and ZAT6 were highly expressed under the low phosphorus environment, reflecting the adaptive regulation of *S. miltiorrhiza* to phosphorus deficiency (Figure 4).

Low-phosphorus treatment activates a variety of adversity response mechanisms, including enhancing nutrient uptake [23], regulating energy metabolism [24], promoting antioxidant responses [25], and regulating root growth [26]. Not only that, *S. miltiorrhiza* saves energy and improves its adaptation to phosphorus-deficient environments by reducing the activity of certain nonessential growth and metabolic processes. Under low phosphorus conditions, *S. miltiorrhiza* inhibits excessive growth and energy consumption by down-regulating the expression of genes such as bHLH20, FAD2, and ERF115, and concentrates its resources to cope with the challenge of phosphorus deficiency; therefore, the low expression of these genes in low phosphorus environments is one of the strategies for plants to adapt to phosphorus-deficient environments [27].

When environmental phosphorus levels are too high, *S. miltiorrhiza* regulates root development and response to various environmental treatments by modulating the expression of bZIP23, NAC, ZAT, and bHLH transcription factors, which improves phosphorus uptake and utilization by the root system and enhances adaptation to environmental stresses [28,29]. CAD genes are highly expressed in high phosphorus environments to enhance lignin synthesis by the plant, which in turn promotes root growth and stress resistance [30]. The up-regulation of SAUR gene expression helps to regulate the growth hormone level of plants, promote the expansion of the root system, and improve the efficiency of phosphorus uptake and utilization in order to adapt to the stressful effects of excess phosphorus on plants [31]. When the phosphorus concentration is too high, *S. miltiorrhiza* reduces fatty acid synthesis by decreasing the expression of FABG1 and instead enhances enzymes or pathways related to phosphorus metabolism to adapt to the state of excess phosphorus resources [32,33].

Similarly, down-regulation of genes encoding TRPD, CML, and CHLP inhibits some non-essential physiological processes, such as pigment synthesis, synthesis of aromatic compounds, and fatty acid biosynthesis, to reduce unnecessary energy consumption and metabolic burden, and to prioritize and optimize phosphorus uptake and utilization [34,35]. At the same time, *S. miltiorrhiza* may regulate the expression of genes related to signaling and protein synthesis to adapt to the stress of adversity in high-phosphorus environments and to ensure the balance of growth and development [36,37], which should be evidenced by the results of KEGG pathway enrichment (Figure 3).

The reasons why the phenylalanine, tyrosine, and tryptophan biosynthesis pathways and the starch and sucrose metabolism pathways of the root system of *S. miltiorrhiza* were highly expressed in both low and high phosphorus environments are mainly related to the adaptive regulatory mechanisms of *S. miltiorrhiza*, the regulation of energy metabolism, the balance of carbon and nitrogen metabolism, and the activation of phosphorus-sensing signaling pathways [38,39]. These pathways regulate the growth and metabolism of *S. miltiorrhiza* in response to different phosphorus environments through the interactions between transcription factors, hormone signaling, and metabolites [40,41].

In the KEGG pathway enrichment analysis performed in the high-phosphorus group, low-phosphorus group, and medium-phosphorus group (Figure 5), among the four pathways co-enriched, arachidonic acid is an evolutionarily conserved signaling molecule involved in regulating the plant stress signaling network and the stress response transcriptional network, which triggers the plant stress and defense signaling network in response to external abiotic adversity [42,43,44]. In addition, abnormal levels of phosphorus also affect the synthesis and transport of phytohormones, the balance of lysine synthesis and degradation, and the activities of enzymes related to nitrogen metabolism and amino acid homeostasis in the root system of *S. miltiorrhiza*, which resists adversity by regulating the above pathways.

By jointly analyzing the transcriptomics and metabolomics data, it was found that the changes in the expression of genes associated with the tanshinone (Figure 6) and salvianolic acid (Figure 7) synthesis pathways were highly consistent with the accumulation pattern of the metabolites. Under P2 and P4 conditions, the expression of MYB and WRKY transcription factors was up-regulated, and the genes for key enzymes in the secondary metabolic pathways associated with them also showed significant up-regulation, which was consistent with the increase in tanshinone and salvianolic acid content. In contrast, the expression of these transcription factors and key enzyme genes was down-regulated under P6 conditions, leading to a decrease in the content of tanshinone and salvianolic acid.

Further analysis showed that abnormal phosphorus supply levels (low and high phosphorus) limited energy metabolism, phosphate synthesis, amino acid metabolism, and phytohormone signaling pathways in the root system of *S. miltiorrhiza*, which, in turn, activated the plant defense mechanisms used against phosphorus treatment. Isoflavonoids, carbohydrate derivatives, unsaturated fatty acids, caffeic acid derivatives, hypoxanthine nucleotides, organic nitrogen-containing compounds, and quinolines were up-regulated in the low phosphorus environment, and these metabolites played important roles in low phosphorus treatment. In contrast, the high expression of isoleucine derivatives, glutathione, tryptophan, and piperidinic acid under the high phosphorus environment reflected the metabolic adaptation of plants.

This study initially revealed the regulatory mechanisms of phosphorus supply level on the growth, development, and secondary metabolism of *S. miltiorrhiza*, but further exploration of the fine regulatory network of phosphorus signaling pathways and their interactions with phytohormones is still needed. Future studies can combine gene editing, protein interactions analysis, and multi-omics integration techniques to deeply analyze the functions of transcription factors (e.g., MYB, WRKY) in the response to phosphorus treatment, as well as the mechanisms regulating the expression of key enzyme genes in the secondary metabolic pathways, so as to provide more comprehensive theoretical support for the molecular breeding of medicinal plants in terms of the efficient utilization of phosphorus and the synthesis of bioactive compounds.

## 4. Materials and Methods

### 4.1. Cultivation of Experimental Materials

The seeds of *S. miltiorrhiza* were obtained from the base of Tianshili in Shangluo City, Shaanxi Province, and were identified as *S. miltiorrhiza* by Prof. Zhang Chenlu from the School of Biological Science and Engineering, Shaanxi University of Technology. Four months after sowing, seedlings of *S. miltiorrhiza* with uniform growth and similar morphology, main root length of about 10 cm, root diameter of about 0.30~0.35 cm, no obvious lateral roots, fresh reeds, and complete red epidermis were selected as the experimental materials, and were transplanted to sterile substrate soil (without fertilizer) for artificial light cultivation for 1 month, and different phosphorus supply levels were set up: of 0 mmol·L^−1^ (P0), 0.0156 mmol·L^−1^ (P1), 0.0313 mmol·L^−1^ (P2), 0.313 mmol·L^−1^ (P3), 0.625 mmol·L^−1^ (P4), 1.25 mmol·L^−1^ (P5), 2.5 mmol·L^−1^ (P6), 5 mmol·L^−1^ (P7), and 10 mmol·L^−1^ (P8) in a series of concentration gradients of phosphorus-deficient total nutrient solution for treatment. The phosphorus-deficient total nutrient solution formulations were based on the modified Hoagland formulation with slight modifications (Tables S6 and S7), with P0, P1, and P2 as the low-phosphorus group, P3, P4, and P5 in the medium-phosphorus group, and P6, P7, and P8 in the high-phosphorus group, and with 0 mmol·L^−1^ (P0) as the blank control group. The samples were collected once every 7 days following each phosphorus treatment. In total, four sampling events were conducted, resulting in four distinct batches of samples. Each batch corresponded to a specific sampling time point and included three replicates for each phosphorus treatment group. Specifically, three seedlings were sampled and analyzed for each treatment group in every batch (Table S8).

### 4.2. Determination of Growth and Development Indexes of S. miltiorrhiza Seedlings

After sampling four batches of *S. miltiorrhiza* seedlings subjected to different phosphorus treatments, we conducted a phenological morphology analysis of the aboveground stem and leaf parts, as well as the underground root parts. The fresh mass of leaves and roots was measured immediately after sampling. Subsequently, the fresh leaves and roots were dried under controlled conditions, and the dry mass of both leaves and roots was determined. These measurements allowed us to compare the developmental state of *S. miltiorrhiza* seedlings under different treatments by analyzing their growth and development indices. The results of these measurements are presented in Figure 1.

### 4.3. Detection of Bioactive Compounds in S. miltiorrhiza Seedlings

#### 4.3.1. *S. miltiorrhiza* Pre-Seedling Treatment

The roots and leaves of *S. miltiorrhiza* seedlings were separated, killed at 105 °C for 15 min, dried at 50 °C, weighed, and pulverized. Samples of precisely 0.100 g were added to 25 mL of 75% methanol solution, weighed, ultrasonicated for 30 min, and centrifuged for 15 min (10,000 r/min). The appropriate supernatant was taken over 0.22 μm microporous filter membrane to complete the pre-test sample processing.

#### 4.3.2. Chromatographic Conditions

The chromatographic column was a SymMeyryC18 (4.6 × 250 mm, 5 μm), and the mobile phases were acetonitrile in A and 0.02% phosphoric acid solution in B at a flow rate of 1 mL/min, with detection wavelengths of 286 nm (for salvianolic acid analogues) and 270 nm (for tanshinone analogues) at a column temperature of 30 °C and injection volume of 10 μL for HPLC on-line detection. The HPLC detection elution program for active metabolites of *S. miltiorrhiza* is shown in Table S9.

#### 4.3.3. Preparation of *S. miltiorrhiza* Control Solution

In order to detect the changes in the content of active metabolites in the roots of *S. miltiorrhiza* seedlings under different levels of phosphorus supply treatment, a total of 11 active metabolites in Salvia roots were compared for differences in content. A standard solution of danshensu, caffeic acid, protocatechuic aldehyde, rosemarinic acid, lithospermic acid, salvianolic acid A, salvianolic acid B, dihydrotanshinone, cryptotanshinone, tanshinone I, and tanshinone IIA was prepared by dissolving in 75% methanol. The standard solution was sourced from Shanghai YuanYe Biotechnology Co., Ltd. (Shanghai, China). The mass concentrations of the standard solution were 1.00 mg·mL^−1^, 1.00 mg·mL^−1^, 1.04 mg·mL^−1^, 0.81 mg·mL^−1^, 1.00 mg·mL^−1^, 0.98 mg·mL^−1^, and 0.84 mg·mL^−1^ for the respective compounds. The standard curves and correlation regression coefficients of the 11 bioactive compounds of *S. miltiorrhiza* detected by HPLC are shown in Table S10, and the correlation regression coefficients of the 11 bioactive compounds of *S. miltiorrhiza* were all >0.999, indicating that the regression equations were reliable.

### 4.4. Transcriptomic Analysis of the Root System of S. miltiorrhiza Seedlings

To analyze and compare the differences in appearance and morphology, secondary metabolite contents, and phosphorus treatment duration of *S. miltiorrhiza* plants from different experimental treatments, several groups of *S. miltiorrhiza* seedlings with large differences in batch 4 were selected for transcriptomics analysis, namely, P0, P2, P4, and P6, and three *S. miltiorrhiza* seedlings with similar growth were selected as three replicates of samples for each group, for a total of 12 samples. Total RNA from the root systems of *S. miltiorrhiza* seedlings of each experimental treatment was extracted by RNAprep Pure Plant Plus Kit (TIANGEN, Beijing, China) Polysaccharide and Polyphenol Plant Total RNA Extraction Kit. RNA quality was assessed using an Agilent 2100 Bioanalyzer (Agilent Technologies, Santa Clara, CA, USA), and RNA integrity values > 6.0 were considered high-quality samples. RNA purity was identified and quantified using a Nano Drop 2000 spectrophotometer (Thermo Scientific, Waltham, MA, USA). The transcriptome libraries were constructed using the VAHTS Universal V5 RNA-seq Library Prep kit according to the instructions, and sequenced using the Illumina Hiseq 2000 sequencing platform. The library construction and transcriptome sequencing were performed by Beijing Novogene Bioinformatics Technology Co., Ltd. (Beijing, China). All samples were transcriptome-sequenced using *S. miltiorrhiza* (GCA_016432925.1_NRC_Smil_1.0) as the reference genome. Samples from the P0 group were used as the control, and differentially expressed metabolites and genes were screened for the P2, P4, and P6 treatments. For data processing, Hisat2 was used for alignment, StringTie for assembly, and clusterProfiler for functional annotation and analysis. Differentially expressed metabolites (DEMs) and differentially expressed genes (DEGs) were screened under the conditions of |log2 (Fold Change) | ≥ 1 and q < 0.05, based on GO and KEGG databases.

### 4.5. Analysis of Expression Changes of DEGs Related to the Synthetic Pathway of Tanshinone and Salvianolic Acid

Based on the results of transcriptome sequencing, differentially expressed genes (DEGs) related to the synthesis pathways of tanshinone and salvianolic acid, including genes encoding key enzymes as well as transcription factors, were screened and combined with differential expression analysis to explore their expression changes and their regulatory roles under different phosphorus treatment conditions. The screening conditions were |log2(Fold Change) | ≥ 1 and q < 0.05 to ensure that the screened genes had significant expression changes under different phosphorus supply levels, and the expression changes of transcription factors (TFs) related to the synthesis pathways of tanshinone and salvianolic acid were further analyzed.

### 4.6. Real-Time Fluorescence Quantitative PCR (qRT-PCR) Gene Validation

To verify the accuracy of gene expression data obtained by RNA-seq, eight genes related to phosphate uptake and transport, bioactive compound accumulation and root growth and development were selected for qRT-PCR validation. Three seedlings of *S. miltiorrhiza* with similar growth conditions were selected from each of the four subgroups of P0, P2, P4, and P6 as three replicates, and the extraction of total RNA was accomplished by using the polysaccharide and polyphenol plant total RNA extraction kit (DP441) from Tiangen Biochemical Technology Co. RNAs of good quality and integrity that were qualified by the assay were reverse transcribed into cDNAs by using a reverse transcription kit provided by Novozymes Biotechnology Co. The cDNA was finally diluted to 50 ng/μL, and specific primers (Table S11) were designed for qRT-PCR using NCBI Primer-BLAST (https://www.ncbi.nlm.nih.gov/tools/primer-blast/ [accessed on 14 December 2024]). Actin was selected as the internal reference gene, and the gene expression analysis was carried out using the 2^−ΔΔCt^ data analysis method to calculate the relative gene expression.

### 4.7. Statistical Analyses

SPSS 22.0, Origin 2021, Adobe Illustrator 2021, GraphPad Prism 9.5, and other graphing software packages were used to analyze the data, and Duncan’s new complex polar deviation method was applied to compare the biomass and phosphorus content of each part of *S. miltiorrhiza* seedlings among different treatments at the α = 0.05 level for significant differences. The P0 treatment was used as the control, and the differentially expressed metabolites (DEMs) and differentially expressed genes (DEGs) were screened in the P2, P4, and P6 treatments, respectively. Conditions were |log2 (Fold Change) | ≥ 1 and q < 0.05, and the DEMs and DEGs were functionally annotated and analyzed based on GO and KEGG databases.

## 5. Conclusions

This study explored the regulatory effects of different phosphorus supply levels on the growth and development of *S. miltiorrhiza* seedlings and the accumulation of bioactive compounds through combined transcriptomics and metabolomics analysis. The biomass of *S. miltiorrhiza* reached its maximum at a phosphorus concentration of 0.625 mmol·L^−1^. Concentrations lower or higher than this level resulted in reduced biomass and inhibited growth and bioactive compound accumulation. We identified 14 and 20 DEGs associated with tanshinone and salvianolic acid synthesis, respectively. Transcription factors like SmCPS1 and SmCYP76AH3 were positively correlated with synthesis pathway genes under low-to-moderate phosphorus conditions but down-regulated at high phosphorus levels. These findings enhance our understanding of phosphorus-regulated growth and secondary metabolism in *S. miltiorrhiza* and provide potential targets for molecular breeding. Overall, this study improves our understanding of phosphorus-regulated growth and metabolism in *S. miltiorrhiza*, offering a foundation for its cultivation and breeding and advancing our knowledge of its molecular regulation.

## Data Availability

The original contributions presented in this study are included in the article/Supplementary Material. Further inquiries can be directed to the corresponding author.

## Supplementary Materials

The following supporting information can be downloaded at: https://www.mdpi.com/article/10.3390/ijms26136253/s1.
