# Supplementary material for: Transcriptome- and Metabolome-Based Regulation of Growth, Development, and Bioactive Compounds in Salvia miltiorrhiza (Lamiaceae) Seedlings by Different Phosphorus Levels
Source: Int J Mol Sci. 2025 Jun 28;26(13):6253. doi: 10.3390/ijms26136253 (PMC12249955; doi:10.3390/ijms26136253)
Supplement: Supplementary file 1 [file ijms-26-06253-s001.zip › Supplementary Figure S1.pdf]

Supplementary Figure S1

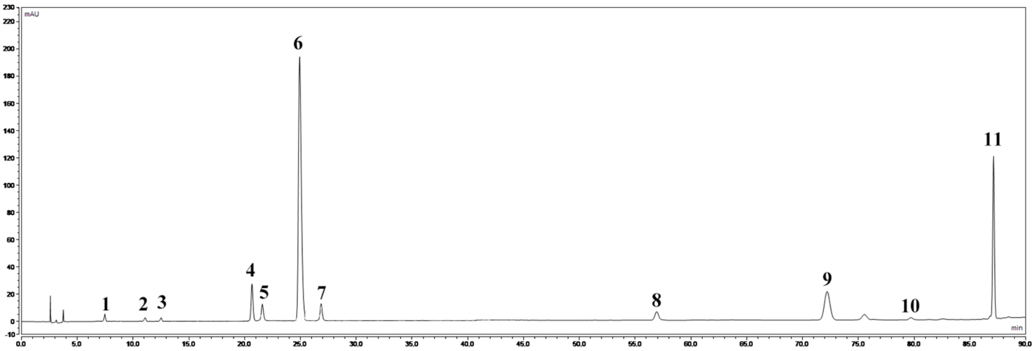

Supplementary Figure S1. HPLC chromatogram of *S. miltiorrhiza* bioactive compound mixed control product.

Based on the retention times of different standards in HPLC, the compounds can be identified as follows: 1 is danshensu, 2 is protocatechuicaldehyde, 3 is caffeic acid, 4 is rosmarinic acid, 5 is lithospermic acid, 6 is salvianolic acid B, 7 is salvianolic acid A, 8 is dihydrotanshinone, 9 is cryptotanshinone, 10 is tanshinone I, and 11 is tanshinone IIA. Among them, the peak height and area of salvianolic acid B are the largest among all the standards.
