# Supplementary material for: Transcriptome- and Metabolome-Based Regulation of Growth, Development, and Bioactive Compounds in Salvia miltiorrhiza (Lamiaceae) Seedlings by Different Phosphorus Levels
Source: Int J Mol Sci. 2025 Jun 28;26(13):6253. doi: 10.3390/ijms26136253 (PMC12249955; doi:10.3390/ijms26136253)
Supplement: Supplementary file 1 [file ijms-26-06253-s001.zip › Supplementary Table S1-S11.pdf]

Supplementary Table S1-S11

Supplementary Table S1. Raw data collation and quality assessment

Sample ID	Raw reads	Clean reads	Raw bases （Gb）	Total map（%）	Q20(%)	Q30(%)	GC pct （%）
P0_a	41875214	40280106	5.78	30845685(80.58%)	97.25	92.33	53.18
P0_b	41234568	39784210	5.98	32416789(81.48%)	97.42	93.02	52.94
P0_c	40567129	39024582	5.88	30123456(77.21%)	97.18	92.33	54.07
P2_a	41042420	39612852	5.94	32586122(82.26%)	97.43	93.14	53.01
P2_b	43454924	41409636	6.21	34083060(82.31%)	97.12	92.48	52.73
P2_c	44820574	43503552	6.53	30680281(70.52%)	97.36	92.94	54.22
P4_a	41152776	40166478	6.02	31120689(77.48%)	97.42	93.08	53.39
P4_b	42294822	40808744	6.12	33281180(81.55%)	97.54	93.3	52.76
P4_c	43608932	41251468	6.19	34257007(83.04%)	97.58	93.38	53.87
P6_a	42931606	41493412	6.22	35346708(85.19%)	97.33	92.77	52.75
P6_b	43293404	41023518	6.15	33697839(82.14%)	97.42	93.05	53.77
P6_c	44869566	40738416	6.11	30544282(74.98%)	97.15	93.15	53.50

Phosphorus treatments were applied at four concentrations: P0 (0.0 mmol·L^{-1}), P2 (0.0313 mmol·L^{-1}), P4 (0.625 mmol·L^{-1}), and P6 (2.5 mmol·L^{-1}). Three biologically independent *S. miltiorrhiza* seedlings with comparable phenotypic traits per treatment were designated as replicates (a, b, c), yielding 12 experimental units for multi-omics analyses.

Supplementary Table S2. Statistical results for differentially expressed genes

Compare	All	Up	Down	Threshold
P0vsP2	1539	775	764	DESeq2padj<=0.05|log2FoldChange|>=1.0
P0vsP4	1889	731	1158	DESeq2padj<=0.05|log2FoldChange|>=1.0
P0vsP6	3053	1525	1528	DESeq2padj<=0.05|log2FoldChange|>=1.0

This table shows the statistical results for differentially expressed genes (DEGs) among different groups (P0, P2, P4, P6). The comparison groups include P0vsP2, P0vsP4, and P0vsP6. The “All” column indicates the total number of differentially expressed genes, The “Up” column indicates the number of up-regulated genes, and the “Down” column indicates the number of down-regulated genes. The screening criteria for differentially expressed genes were DESeq2-adjusted p-value (padj) ≤ 0.05 and log2-fold change (FoldChange) ≥ 1.0 in absolute value.

Supplementary Table S3. GO analysis of up-regulated differentially expressed genes

	BP	CC	MF
P0vsP2	translation	ribosome	ion transmembrane transporter activity
	amide biosynthetic process	ribonucleoprotein complex	structural molecule activity
	peptide biosynthetic process	non-membrane-bounded organelle	GTPase activity
	peptide metabolic process	intracellular non-membrane-bounded organelle	/
	cellular amide metabolic process	cytoplasmic part	/
P0vsP4	amide biosynthetic process	non-membrane-bounded organelle	structural constituent of ribosome
	nucleosome assembly	intracellular non-membrane-bounded organelle	heme binding
	chromatin assembly	nucleosome	/
	nucleosome organization	protein−DNA complex	/
	protein−DNA complex assembly	DNA packaging complex	/
	DNA packaging	ribosome	/
P0vsP6	cellular ketone metabolic process	oxidoreductase complex	DNA-binding transcription factor activity
	defense response	organelle inner membrane	transferase activity
	amide biosynthetic process	/	structural constituent of ribosome
	cellular amide metabolic process	/	transcription regulator activity
	translation	/	structural molecule activity

BP, MF, and CC are the three main classifications that stand for Biological Process (BP), Molecular Function (MF) and Cellular Component (CC), respectively. Together, they are used to describe the function and action of genes or proteins.

Supplementary Table S4. GO analysis of down-regulated differentially expressed genes

	BP	CC	MF
P0vsP2	carbohydrate metabolic process	extracellular region	hydrolase activity, hydrolyzing O-glycosyl compounds
	defense response	chromatin	carbohydrate binding
	cellular carbohydrate metabolic process	cell wall	copper ion binding
	small molecule biosynthetic process	external encapsulating structure	active transmembrane transporter activity
	drug transport	apoplast	hydrolase activity, acting on glycosyl bonds
	drug transmembrane transport	cell periphery	serine-type peptidase activity
P0vsP4	monovalent inorganic cation transport	Golgi-associated vesicle	oxidoreductase activity, acting on paired
	intracellular protein transport	Golgi apparatus part	N, N-dimethylaniline monooxygenase activity
	metal ion transport	membrane coat	GTPase binding
	intracellular transport	coated membrane	N-acyltransferase activity
	establishment of localization in cell	bounding membrane of organelle	/
	carbohydrate metabolic process	/	/
P0vsP6	hexose metabolic process	coated membrane	NADP binding
	monosaccharide metabolic process	cell cortex	oxidoreductase activity
	glucose metabolic process	cell cortex part	active transmembrane transporter activity
	carbohydrate metabolic process	cytoplasmic region	coenzyme binding
	small molecule catabolic process	/	oxidoreductase activity, acting on CH−OH
	response to chemical	/	vitamin binding

BP, MF, and CC are the three main classifications that stand for Biological Process (BP), Molecular Function (MF) and Cellular Component (CC), respectively. Together, they are used to describe the function and action of genes or proteins.

Supplementary Table S5. KEGG analysis of differentially expressed genes

	UP	DOWN
P0vsP2	ubiquinone and other terpenoid−quinone biosynthesis	starch and sucrose metabolism
	metabolic pathways	galactose metabolism
	aminoacyl−tRNA biosynthesis	diterpenoid biosynthesis
	phenylalanine metabolism	biosynthesis of secondary metabolites
	folate biosynthesis	ABC transporters
	/	zeatin biosynthesis
P0vsP4	biosynthesis of flavonoids and flavonols	lysine degradation
	tyrosine	fructose and mannose metabolism
	pyrimidine metabolism	amino sugar and nucleotide sugar metabolism
	tryptophan metabolism	terpenoid backbone biosynthesis
	/	phosphonate and phosphate metabolism
P0vsP6	biosynthesis of amino acids	pentose phosphate pathway
	biotin metabolism	oxidative phosphorylation
	arginine biosynthesis	phenylalanine metabolism
	cysteine and methionine metabolism	alanine, aspartate and glutamate metabolism
	vitamin B6 metabolism	citrate cycle (TCA cycle)
	glyoxylate and dicarboxylate metabolism	/

This table shows the results of KEGG pathway enrichment analysis of differentially expressed genes between different groups (P0, P2, P4, P6). The comparison groups include P0vsP2, P0vsP4, and P0vsP6. The “UP” column indicates KEGG pathways enriched by up-regulated genes, and the “DOWN” column indicates KEGG pathways enriched by down-regulated genes. Enrichment analysis helps to reveal the significant enrichment of differentially expressed genes in specific metabolic pathways or signaling pathways, thus providing clues for the study of gene functions and mechanisms.

Supplementary Table S6. Ingredient composition of modified Hoagland nutrient solution

Composition of working fluid	mg/L
$Ca(NO_3)_2·4H_2O$	945
KNO_3	506
NH_4NO_3	80
KH_2PO_4	Calculated based on P level
$MgSO_4$	241
FeNaEDTA	36.7
KI	0.83
H_3BO_3	6.2
$MnSO_4·H_2O$	16.9
$ZnSO_4·7H_2O$	8.6
$Na_2MoO_4·2H_2O$	0.25
$CuSO_4·5H_2O$	0.025
$CoCl_2·6H_2O$	0.025
pH(25°C）	5.8±0.2

This table lists the concentrations (in mg/L) of the components in the modified Hoagland nutrient solution. The formulation of the nutrient solution was based on a variety of mineral elements required for plant growth, of which $Ca(NO_3)_2$-$4H_2O$, KNO_3, NH_4NO_3, etc., were the main nitrogen and nutrient sources. The content of KH_2PO_4 was calculated and adjusted according to the different levels of phosphorus in order to meet the experiment-specific requirements for phosphorus concentration. Other components, such as FeNaEDTA, KI, etc., provided essential trace elements to the plants. The pH of the nutrient solution (at 25°C) was controlled within the range of 5.8 ± 0.2 to ensure effective nutrient uptake and utilization by the plants.

Supplementary Table S7. Calculation of KH_2PO_4 concentration for different phosphorus supply levels

Phosphorus Level（$mmol·L^{-1}$）	KH_2PO_4（mg/L）
0$mmol·L^{-1}$（P0）	0
0.0156$mmol·L^{-1}$（P1）	2.13
0.0313$mmol·L^{-1}$（P2）	4.25
0.313$mmol·L^{-1}$（P3）	42.54
0.625$mmol·L^{-1}$（P4）	85.05
1.25$mmol·L^{-1}$（P5）	170.10
2.5$mmol·L^{-1}$（P6）	340.20
5$mmol·L^{-1}$（P7）	680.40
10$mmol·L^{-1}$（P8）	1360.90

This table shows the calculation of KH_2PO_4 concentration (in mg/L) corresponding to different phosphorus supply levels (in mmol-L^{-1}). In the experimental design, the phosphorus content in the nutrient solution was precisely controlled by adjusting the concentration of KH_2PO_4 in order to study the effects of different phosphorus levels on plant growth and physiological processes. The different phosphorus level gradients from P0 (0 mmol-L^{-1}) to P8 (10 mmol-L^{-1}) in the table provide diverse phosphorus supply conditions for the experiments, which can help to analyze in depth the role of phosphorus in plant growth and development and its related mechanisms.

Supplementary Table S8. Sampling schedule for *S. miltiorrhiza*

Time	Phosphorus stress	Deionized water irrigation	Sampling
2022.12.08	First phosphorus treatment	/	/
2022.12.11	/	Normal watering	/
2022.12.15	/	/	First sampling
2022.12.16	Second phosphorus treatment	/	/
2022.12.19	/	Normal watering	/
2022.12.23	/	/	Second sampling
2022.12.24	Third phosphorus treatment	/	/
2022.12.27	/	Normal watering	/
2022.12.31	/	/	Third sampling
2023.01.01	Fourth phosphorus treatment	/	/
2023.01.04	/	Normal watering	/
2023.01.08	/	/	Fourth sampling

Table S8 outlines the experimental timeline for examining the effects of phosphorus stress on *S. miltiorrhiza*. It includes the dates of phosphorus stress treatments, normal deionized water irrigation, and sample collection. The table helps track the timing of these activities, showing how stress treatments and watering were alternated and when samples were taken for analysis.

Supplementary Table S9. Content detection conditions for HPLC

T/min	A/%	B/%
0.000	5.0	95.0
10.000	15.0	85.0
15.000	25.0	75.0
20.000	25.0	75.0
35.000	32.0	68.0
45.000	55.0	45.0
50.000	49.0	51.0
70.000	52.0	48.0
82.000	65.0	35.0
87.000	100.0	0.0
95.000	5.0	95.0

This table outlines the HPLC gradient elution program used for analyzing the active components in *S. miltiorrhiza*. In this setup, A represents acetonitrile, and B represents 0.02% phosphoric acid solution. The table details the percentage composition of A and B over specific time intervals during the HPLC run.

Supplementary Table S10. Standard curve of HPLC detection of bioactive compounds in *S. miltiorrhiza*

Active ingredient	Retention period (min)	Linear range μg/mL	Standard curve	Correlation coefficient
Danshensu	7.490	0.0545-0.3270	Y=5.097x-0.0445	R^2=0.9995
Protocatechuic aldehyde	11.061	0.0038-0.0228	Y=70.49x-0.0732	R^2=0.9993
Caffeic acid	12.571	0.0026-0.0156	Y=27.875x+0.0067	R^2=0.9991
Rosmarinic acid	20.663	0.1130-0.6780	Y=18.197x-0.2222	R^2=0.9993
Lithospermic acid	21.693	0.0530-0.3180	Y=24.402x-0.0693	R^2=0.9990
Salvianolic acid B	25.183	0.0200-0.3200	Y=8.9622x-0.0589	R^2=0.9992
Salvianolic acid A	27.137	0.0500-0.3000	Y=20.195x+0.1542	R^2=0.9994
Dihydrotanshinone	56.760	0.0158-0.0948	Y=97.118x-0.9188	R^2=0.9990
Cryptotanshinone	72.027	0.0564-0.3384	Y=19.963x-0.1583	R^2=0.9992
Tanshinone I	79.573	0.0920-0.5520	Y=29.23x+0.2151	R^2=0.9994
Tanshinone IIA	87.057	0.0840-0.5040	Y=1.0802x-0.0442	R^2=0.9991

This table shows the parameters of the standard curve for the determination of the active ingredients in *S. miltiorrhiza* by high performance liquid chromatography (HPLC). Among them, the "Active Ingredients" column lists the active ingredients of Danshen detected; "Retention Time (min)" indicates the peak appearance time of each active ingredient in HPLC detection; "Linear Range (μg/mL) is the concentration interval in which the concentration of each active ingredient is linearly related to the detection signal; "Standard curve" provides the linear regression equation for quantitative analysis; "Correlation coefficient (R^2)" reflects the goodness of fit of the linear relationship. The closer the value is to 1, the better the linear relationship is.

Supplementary Table S11. The corresponding primers for qRT-PCR

Gene name	Accession number	Primer sequence (5'-3')
WRKY6F	LOC131010795	AGCCGTTTGTGGAAAAGGGA
WRKY6R		AGCCAAACAAGTTTTACTCTGAATG
MYB75F	LOC131001051	CACGACAAACCCCAAGTGTG
MYB75R		GAGAGAGCTGTTGTCGGAGG
HMGR3F	LOC131020849	GGCGTGGAAAATCAGTTGTATG
HMGR3R		ACCAAGAGCACCAGCAATAG
bZIP23F	LOC131010826	TGGCACCCTGTGAATAGCAG
bZIP23R		GCTGTGGAGCAGACCTTACA
CYP71D375F	LOC130990203	CCGACCTCTATCCTTCCGTC
CYP71D375R		AGGTAATTCAGTCCCATCTCGT
PAL4F	LOC131025331	GAAGATCTGGACGCGCTAGT
PAL4R		CGAGGTGGAGCCGTTACTG
ERF115F	LOC131008608	AGACACTACAGAGGAGTCCGT
ERF115R		GTTGAGCTTTGCCTTGTGGC
CPS1F	LOC131015110	AAGGGAGTTTTCAGTGGGAGA
CPS1R		CGACTCGATCATCTGCCCG
Actin F	/	AGGAACCACCGATCCAGACA
Actin R		GGTGCCCTGAGGTCCTGTT

This table lists information about the primers used for the qRT-PCR assay. It includes the gene name, the corresponding gene accession number, and the primer sequence (5' - 3' direction). These primers are used to specifically amplify the target gene for gene expression analysis. The table contains several genes related to plant physiological processes and their corresponding forward (F) and reverse (R) primer sequences, as well as the primer sequence of β-Actin as an internal reference gene, which is used for the standardization and calibration of experimental data.
